# Developing a Web-Based Advisory Expert System for Implementing Traffic Calming Strategies

**DOI:** 10.1155/2014/757981

**Published:** 2014-09-07

**Authors:** Amir Falamarzi, Muhamad Nazri Borhan, Riza Atiq O. K. Rahmat

**Affiliations:** ^1^Sustainable Urban Transport Research Centre (SUTRA), Faculty of Engineering, Universiti Kebangsaan Malaysia, 43600 Selangor Darul Ehsan, Malaysia; ^2^Department of Civil & Structural Engineering, Faculty of Engineering, Universiti Kebangsaan Malaysia, 43600 Selangor Darul Ehsan, Malaysia

## Abstract

Lack of traffic safety has become a serious issue in residential areas. In this paper, a web-based advisory expert system for the purpose of applying traffic calming strategies on residential streets is described because there currently lacks a structured framework for the implementation of such strategies. Developing an expert system can assist and advise engineers for dealing with traffic safety problems. This expert system is developed to fill the gap between the traffic safety experts and people who seek to employ traffic calming strategies including decision makers, engineers, and students. In order to build the expert system, examining sources related to traffic calming studies as well as interviewing with domain experts have been carried out. The system includes above 150 rules and 200 images for different types of measures. The system has three main functions including classifying traffic calming measures, prioritizing traffic calming strategies, and presenting solutions for different traffic safety problems. Verifying, validating processes, and comparing the system with similar works have shown that the system is consistent and acceptable for practical uses. Finally, some recommendations for improving the system are presented.

## 1. Introduction

Nowadays, along with the development of urbanism and an increasing number of vehicles, urban streets, and especially residential streets, suffer from different traffic safety problems [1]. Speeding, thru traffic, and other safety-related problems increase the risk of collisions between pedestrians and vehicles. Additionally, excessive numbers of vehicles in residential streets have lowered the quality of environmental factors in residential areas such as air and noise pollution. Furthermore, motorized transportation and lack of infrastructure can affect the perception of other road users toward the function of streets [2]. For example, traffic congestion and narrow sidewalks can prevent pedestrians from walking as a means of transportation and, consequently, persuade them to use private vehicles [3].

The main purposes of traffic calming strategies are to reduce the speed of vehicles and the amount of nonlocal traffic volume entering residential streets [4]. Traffic calming strategies are designed to make streets safe and calm for nonmotorized transportation users including pedestrians, residents, and children [5]. They consist of physical and nonphysical engineering measures. Speed humps, chicanes, and traffic circles are common physical traffic calming measures. Speed limit reduction and installing signs that prohibit turning are examples of nonphysical traffic calming measures [6]. Traffic calming strategies have a great impact on the safety of residential streets, the effect of which, in treated streets is highly dependent on the location of implementations, space between measures, and design considerations [7]. However, it has been proven that the impact of major traffic safety problems such as speeding and thru traffic have been mitigated in residential streets after implementations have been made.

Employing traffic calming strategies to deal with safety-related problems in residential streets requires experience and knowledge which can be achieved from traffic calming manuals and experts. Due to a wide range of strategies as well as problems in this field, deriving appropriate solutions and effective mechanisms in traffic calming studies is essential. Creating a framework for implementing traffic calming strategies can help both novice and experienced engineers to better recognize problems and accordingly apply appropriate solutions. Expert systems are an interesting branch of artificial intelligence (AI) that are designed and structured to facilitate the decision making process for nonexperts or novice engineers. Expert systems are computer-based programs which are developed to mimic and imitate problem-solving processes along with the reasoning of human experts in different knowledge fields [8–11]. Expert systems can assist humans in solving problems that require extensive knowledge or huge amounts of time. These systems are also applicable in dealing with problems related to computer science, agriculture, nutrition, medicine, engineering, education, geology, and so forth. One of their main advantages is that they are easy to access through computer technology [12].

Useful expert systems have already been developed in the field of transportation and safety.* USLIMITS2* is a web-based expert system which aims to assist engineers in the selection of safe and appropriate speed limits in speed zones on all American roads [13].* Paver* is a knowledge-based expert system developed for the management, maintenance, and rehabilitation of pavement.* Paver* is applicable to military installations, municipalities, airports, researchers in universities, and for the use of consultant companies [14].* COPRBU* is a knowledge-based expert system developed to deal with problems relating to public buses with respect to routes and schedule, level of service, and their reliability. Wen [15] designed an automatic and dynamic expert system for solving congestion problems at traffic lights. Logi and Ritchie [16] came up with a real-time knowledge-based system for managing and controlling traffic congestion within road networks. Castro et al. [17] developed a fuzzy expert system for forecasting collisions between pedestrians and vehicles in an attempt to avoid accidents. E-ASSIST is an expert system designed to assist engineers and decision makers in implementing TDM strategies. In this expert system, TDM strategies are classified and organised. Hence, users are enabled to find the strategies effectively based on the domain knowledge [18].

It has been proven that web-based expert systems can take on an important role in spreading knowledge among engineers and researchers because they are accessible anywhere and at any time, only requiring an internet connection with no installation needed [19]. Furthermore, knowledge or solutions proposed by noncommercial expert systems can be delivered to users without any middlemen [20]. One of the valuable benefits of web-based expert systems is having the potential to be assessed globally through the internet. Also, developers of web-based expert systems are able to monitor the number of visitors and analyze their online feedback.

In the following, Section 2 addresses the problem statement of the study.  Section 3 discusses the importance of the proposed expert system.  Section 4 describes the development process of the expert system including knowledge acquisition, selection of building tool, knowledge representation, CALMSYS structure, knowledge base, and user interface.  Section 5 describes the evaluation of the system which consists of system verification, system validation, and comparison with similar works.  Section 6 provides the conclusion of the study and takes a look at future work which can strengthen the performance of the expert system.

## 2. Problem Statement

Speeding and nonlocal traffic in residential streets are major safety problems in residential streets. Conventional traffic calming manuals normally deal with only speeding and thru traffic, while traffic calming strategies have the ability and potential to handle wider ranges of traffic safety problems in residential streets. Safety parameters such as the condition of nonmotorized transportation users, geometric design, public transportation, lack of infrastructure, special zones, and development factors are all absent in the decision making process of current traffic calming manuals and standards. Furthermore, the classifications of current traffic calming strategies are not well organized because they do not cover all available measures. In this regard, some measures are neglected and some strategies are not developed.

## 3. The Importance of CALMSYS

An advisory expert system can be developed firstly to classify strategies and solutions in traffic calming subject. Strategies can be classified in physical, nonphysical, and combined categories. Policy making, psychological measures, traffic restriction and prevention, infrastructure improvement, and enforcement are suitable strategies which can be categorized in traffic calming studies. More importantly, an advisory expert system has the ability to assist engineers in finding proper traffic calming strategies toward traffic safety problems. In this regard, users and engineers will be helped to employ proper solutions to related safety problems. Hence, this expert system has included all possible traffic safety problems which can be solved by employing traffic calming strategies. Moreover, designing traffic calming measures and also ranking traffic calming projects can be handled better and more successfully by computerized systems than by human experts due to the presence of large numbers of data and complex mathematical equations.

## 4. Development of CALMSYS

In this section, different parts of the traffic calming expert system are described including knowledge acquisition, knowledge base, building tool, and user interface of CALMSYS.

### 4.1. Knowledge Acquisition

Knowledge for developing a traffic calming expert system can be collected and obtained from human expertise in traffic calming as well as written sources of knowledge. There are various sources of manuals, journals, and books existing in the field of traffic calming that can serve as the knowledge core for the proposed expert system. Written sources in traffic calming studies include different subjects and frameworks, but most of them contain descriptions of traffic calming measures, results from the impact of traffic calming measures, and ranking processes of projects.  Table 1 shows a list of major written sources in this field.

The second source of knowledge for developing expert systems is domain experts. Selecting domain experts is one of the most essential parts of expert system development. Domain experts must be knowledgeable and have adequate experience in the field. The depth of experience and the type of experience (theoretical, practical, or a combination of both) must be taken into account when choosing domain experts to help develop traffic calming advisory expert systems [26]. Domain experts with greater experience and having practical experience are preferred, but it must be noted that involving younger experts who have innovative ideas to tackle complex problems can also be useful. In this study, 15 experts in the field of traffic calming and safety have been asked to identify and explain solutions for dealing with traffic safety problems in residential streets. Experts are divided into three groups. The first group includes young experts with 5 to 10 years of experience. The second group includes experts with experience ranging from 10 to 15 years. The third group includes experts with more than 15 years of experience. The average length of experience of all the experts is 15 years.

### 4.2. Selection of Building Tool

For developing the expert system, Microsoft Visual Basic.NET was used. The main advantage of this version over VB 6.0 is the capability of VB.NET to build a web-based expert system. Generally, Visual Basic software is an easy to learn and flexible language that enables developers to code and create GUIs (graphical user interfaces) [27]. In a graphical user interface environment, users have icons, pictures, menus, and other useful elements which are not provided in basic expert systems shells. An expert system integrated with GUIs can make the system more accessible for users who do not have a high level of proficiency [28]. Furthermore, being simple allows other developers to modify or upgrade expert systems created by Visual Basic software.

### 4.3. Knowledge Representation

For developing the expert system in this study IF-THEN rules have been employed to represent the knowledge base. In total, more than 150 rules were generated. 100 rules were applied in the first module and the other 50 rules were applied in both the second and third modules. IF-THEN rules are an effective and useful type of forward-chaining inference engine where the decision making process is started from the data entered by users and ended with the attainment of a particular goal. In this method, for example, if the problem (A) is true, then the solution (B) will be recommended to users.

### 4.4. CALMSYS Structure

**Figure 1:**
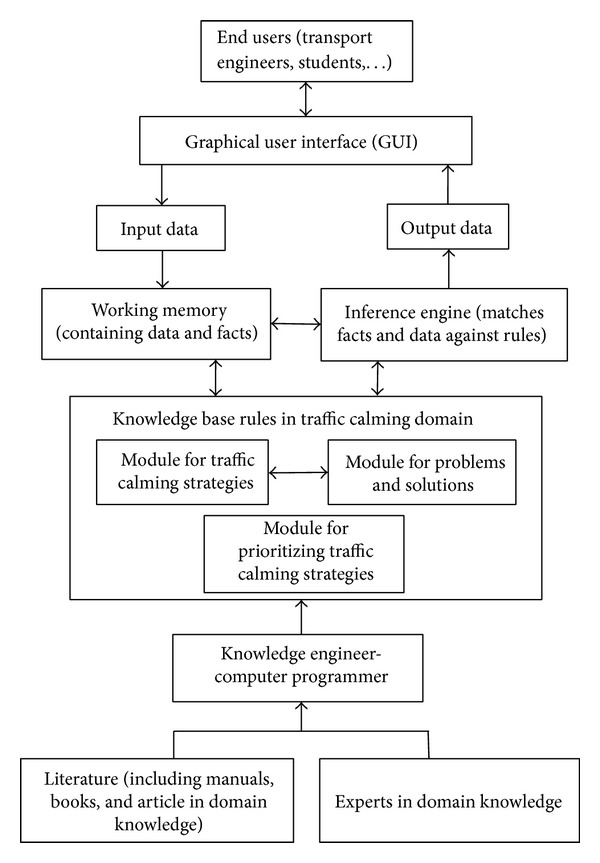
The structure of CALMSYS.

Figure 1 shows the structure of CALMSYS which have consisted of the relations between the main component of the expert system including working memory, inference engine, knowledge base, and user interface.

### 4.5. Knowledge Base

In this study, the traffic calming advisory expert system is composed of three main modules including a module for the classification of traffic calming strategies, a module for problems and solutions, and a module for prioritizing traffic calming strategies. The modules of this expert system can work independently but there is collaboration between the module of traffic calming strategies and the module of problems and solutions which have been described in Section 4.6.2. Descriptions related to the main modules of CALMSYS are provided in the following passages.

#### 4.5.1. Module for Traffic Calming Strategies

An important step before finding solutions for traffic calming problems is to develop and explore traffic calming strategies. For this purpose, the main function of current traffic calming measures was gathered from available sources including traffic calming manuals, related books, and journal articles which are discussed in the knowledge acquisition section of this research paper. Furthermore, consulting with domain experts has improved overall knowledge of the subject and brought successful outcomes in traffic calming studies that have been applied in different places. It must be noted that implementation guidelines, advantages, disadvantages, and the design process can all enhance the knowledge base of an expert system.

In addition, traffic calming measures must be categorized into specific strategies in order to employ them effectively according to their performance and characteristics. Interviewing with domain experts has been proven to help engineers in the field conduct classifications of traffic calming measures.  Table 2 shows the classification of traffic calming measures according to eleven categories of strategies.

In this module, descriptions of traffic calming strategies, including the measures mentioned in Table 2, have been provided and are available to end-users. In addition to the classification, designs of vertical and horizontal deflections have been included in this module. For example, according to the traffic calming manual published by ITE, there is a direct relation between length of vertical traffic calming measures and their crossing speed (design speed). In chicanes and lateral shifts, path angle has direct relation with the crossing speed of vehicles passing the measures. An example of employing this module is represented in the user interface section.

#### 4.5.2. The Module for Problems and Solutions

There are various safety problems in residential streets that can be treated by employing traffic calming strategies. In this study, elicitation of knowledge from both written sources and domain experts was carried out. Firstly, reviewing and examining written sources of the subject field to find traffic safety problems are essential. Although investigating traffic calming manuals and related books can give a general understanding to engineers about the safety problems that pedestrians and other road-users may face while traveling on residential streets, in most traffic calming manuals, capabilities of traffic calming strategies to solve various traffic safety problems are not defined clearly and only descriptions are presented to readers.

The next step was to pinpoint traffic safety parameters which can cause safety problems in residential streets. To accomplish this, interviewing experts in the domain can be useful because these types of experts generally have a good deal of practical experience in facing these types of traffic safety problems. The duty of safety experts is to find solutions for different traffic safety problems. In this study several interviews with the domain experts were carried out. Experts were asked to express the parameters that they believe have the potential to be included in studies related to traffic calming and safety of residential streets. As a result, a list of the parameters categorized as traffic-related parameters, geometric design parameters, deficiencies in street infrastructure, and land-use related parameters was compiled.

Afterwards, two questionnaires were designed and distributed among the experts. In the first questionnaire, experts were asked to select the importance of each parameter in traffic calming studies, which were elicited through the above methods, according to a 5-point Likert Scale. In this rating scale, 1 means strongly unimportant, 2 means unimportant, 3 means neutral, 4 means important, and 5 means strongly important. Furthermore, in this questionnaire, in the event that experts felt the given parameters could present safety problems and that employing traffic calming is necessary, they were asked to specify the associated threshold and conditions of the related traffic safety parameters. After collecting questionnaires, the data was analysed by* SPSS* software. Mean values and standard values were calculated as shown in Table 3.* ANOVA* was used in order to determine whether the answers of the three groups of experts were significantly different from each other or not. According to the *P* value in Table 3, no significant difference between the groups was found. This means that experts with different lengths of relevant experience have similar notions toward the importance of the traffic safety parameters contained in the expert system.  Table 4 presents the thresholds of traffic safety parameters.

The second questionnaire focuses on the solutions or strategies that experts have applied or recommended for dealing with the mentioned problems. To achieve the purpose, a list of traffic calming strategies with their associated measures and details was presented to them. Then, the experts could choose the measures under each strategy to match the safety problems according to their knowledge and experience. Experts could describe solutions for each problem and propose their own ideas and methods to tackle the problems. Finally, three experts with the highest level of experience were selected to evaluate the answers and finalize the solutions for each traffic safety problem.  Table 5 summarizes the solutions that the domain experts have proposed. As an example, the process of running this module is illustrated in the user interface section.

#### 4.5.3. Module for Prioritizing the Strategies

**Figure 2:**
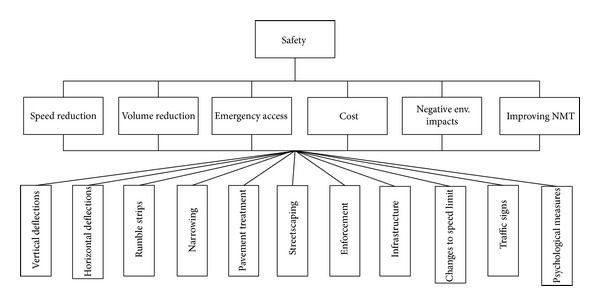
Hierarchical structure of the model.

Functions of traffic calming measures in terms of different criteria including speed reduction, volume reduction, improvement of nonmotorized transportation, environmental impacts, emergency access, and cost of implementation and maintenance must be compared in order to prioritize them. The analytic hierarchy process (AHP) is a multicriteria decision Making (MCDM) tool developed to make a correct decision with regard to the goal and proposed criteria [29]. The AHP technique has been used in a wide range of fields such as project management, software selection, and marketing [30]. The AHP technique can assist engineers in employing traffic calming strategies when considering and integrating different criteria are required. The first step for prioritizing strategies is to create a hierarchical structure of the model as in Figure 2. The goal is located at the top of the model. The second level contains the criteria in prioritizing strategies while the third level is for alternatives or traffic calming strategies.

**Figure 3:**
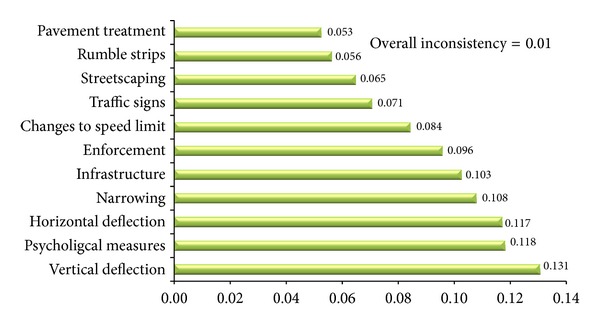
The composite priority weights of traffic calming strategies.

For developing the AHP technique, domain experts selected for knowledge acquisition and developing the expert system were asked to participate in this study. The* Expert Choice* (Version 11) software was used to prioritize traffic calming strategies with respect to different criteria. Due to a large number of calculations, using* Expert Choice* software can facilitate the group decision making process and reduce errors occurred in manual computation. Normalized scores of traffic calming strategies with respect to the different criteria and the weight of each criterion which was obtained from pairwise comparison judgments matrices were calculated and determined. Finally, the composite priority weights of traffic calming strategies were determined as shown in Figure 3. The overall consistency of the model is 0.01 which indicates that the judgments of experts are carried out correctly. In this module end-users are enable to compare traffic calming strategies with respect to the criteria and employ them according to their needs. An example of running this module is provided in the user interface section.

### 4.6. User Interface

**Figure 4:**
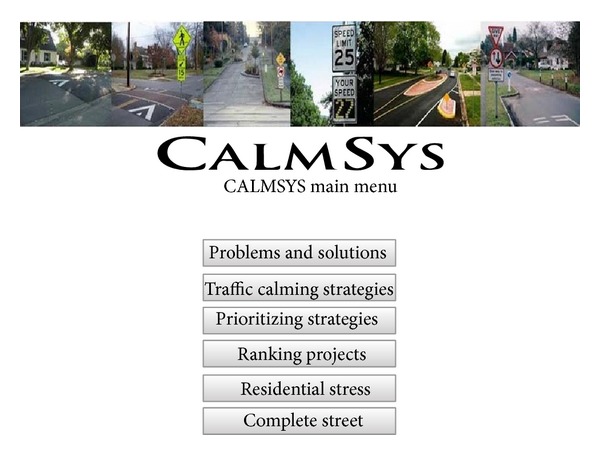
A screenshot of the CALMSYS main menu.

Making expert systems as user-friendly as possible and avoiding creating a complicated design can attract users to utilize expert systems in their fields. In advisory expert systems, users must be able to find their problems without being confused or easily frustrated from difficult procedures. The CALMSYS web-based expert system has provided different and useful functions for its users. Main modules of the system are displayed as toolboxes in the user interface. In addition, toolboxes for ranking traffic calming projects and developing complete streets are provided for assisting end-users.  Figure 4 shows the main menu screenshot. In this section, the function of toolboxes for traffic calming strategies, problems and solutions, and prioritizing strategies are demonstrated with practical examples.

#### 4.6.1. Toolbox for Traffic Calming Strategies

In the toolbox for traffic calming strategies, different traffic calming measures are classified in terms of the strategies that have been previously described. Users are able to select different traffic calming measures according to their purposes by clicking on them. For each measure, a description, typical example, advantages and design considerations are provided.  Table 6 shows a screenshot of the toolbox for traffic calming strategies.

**Figure 5:**
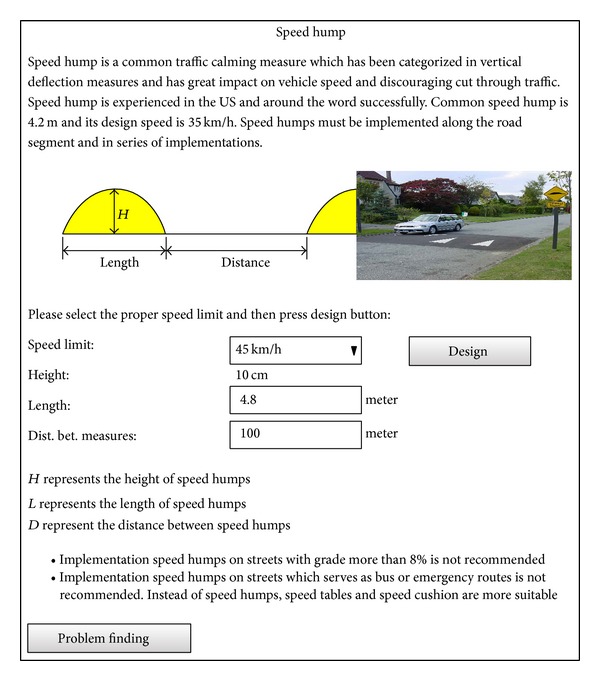
A screenshot of speed hump design.

In this toolbox, there are 52 hyperlinks that can serve as a useful source for engineers and students. For example, by clicking on the hyperlink for speed hump design, the designing process for the measure according to the ITE procedure is detailed (Figure 5). Speed humps are common and effective traffic calming measures which have been categorized in vertical deflection strategies. Their effectiveness on speed reduction and reducing thru traffic is proven. Length of speed humps has an important role on their performance. Shorter lengths and greater heights can slow down vehicles drastically but they are not suitable for collector streets or streets with speed limits above 40 km/h. On this page, when users select the speed limit of the target streets from the dropdown list, the expert system inference engine provides the proper length, height, and distance between the measures accordingly in the dedicated textboxes. For example as shown in Figure 5, selecting speed limit of 45 km/h leads to design of a speed hump with height of 10 cm, length of 4.8 m and distance (between measures) of 100 m. In addition to the proposed geometric design, useful design considerations for implementing speed humps are recommended to users. In this regard, implementing speed humps on streets with bus routes is not recommended. Similarly, on streets with a grade of more than 8 percent implementation can cause accidents.

#### 4.6.2. Toolbox for Problems and Solutions

**Figure 6:**
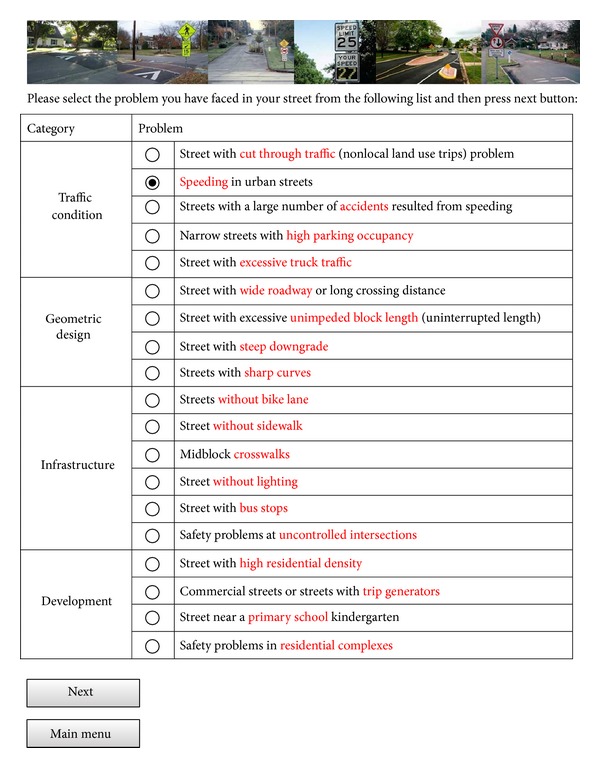
A screenshot of the problems and solutions page.

In the toolbox for problems and solutions, a list of 19 traffic safety problems which were described previously are presented to users as shown in Figure 6. On this page users can select the problems they have faced. Key words of problems are highlighted with different colours which can facilitate the process of finding the right problem for users of the expert system. Descriptions of problems can help users and engineers know the problems and their effect on safety of road users.

**Figure 7:**
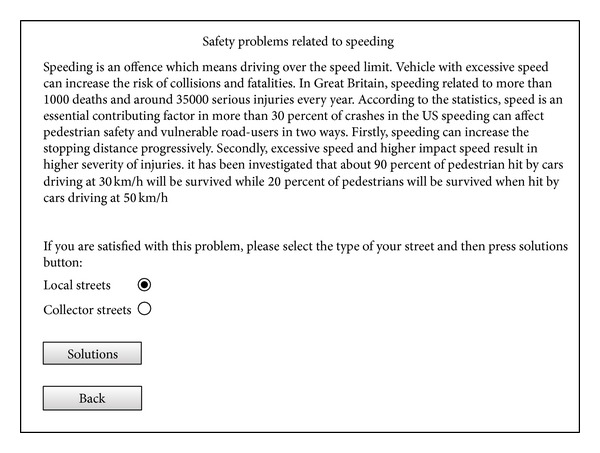
A screenshot of the speeding problems page.

**Figure 8:**
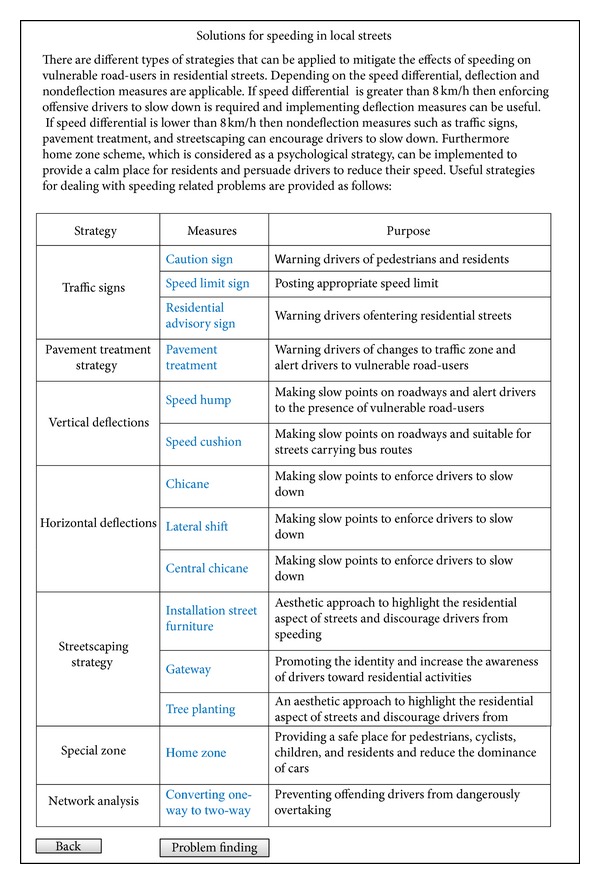
A screenshot of the solutions for speeding page.

For example, if a street with speeding related problems is selected, users will be directed to the speeding problems page as illustrated in Figure 7 which describes the results and negative impacts of speeding in residential streets on road-users. In this page users will be informed that speeding can affect pedestrian safety and vulnerable road-users in two ways. Firstly speeding can increase a vehicle's stopping distance exponentially. Secondly, excessive speed and higher impact speed will result in higher severity of injuries. At the bottom of this page, users have to select the type of street (local or collector streets) and, according to their selection, the solutions which can prevent and reduce the impacts of speeding on residents and other road-users will be presented.  Figure 8 shows a screenshot of the page related to solutions for speeding problems in residential streets.

On the page of solutions for speeding, there are different traffic calming strategies which user can employ to deal with the problem. Traffic signs, pavement treatment, vertical deflections, horizontal deflections, streetscaping, and special zones are useful strategies that can eliminate or reduce problem of speeding in residential streets. It must be mentioned that the toolbox of problems and solutions and the toolbox of traffic calming strategies are connected to each other by means of hyperlinks. These hyperlinks have integrated different parts of the system effectively and facilitated the use of the system. As shown in Figure 8, each strategy have one or more measures that by clicking on them users of the system will be directed to the descriptions or design pages related to the toolbox of traffic calming strategies.

#### 4.6.3. Toolbox for Prioritizing the Strategies

**Figure 9:**
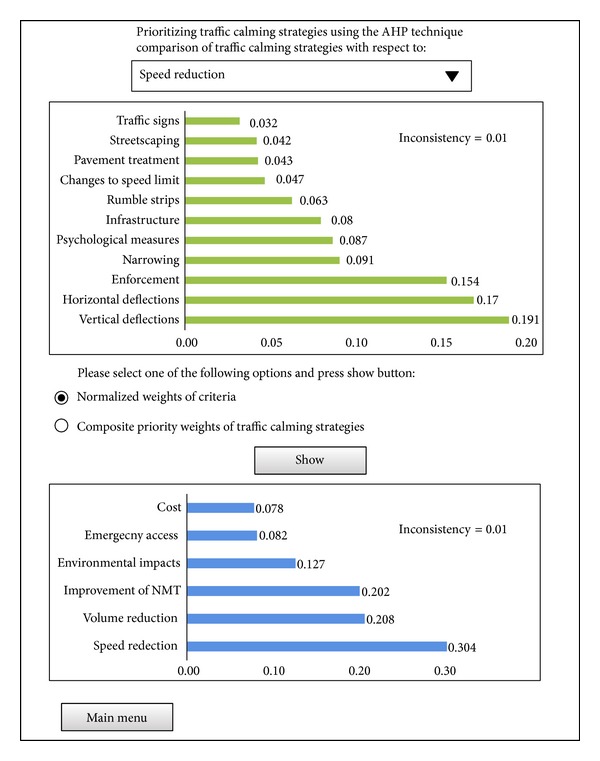
A screenshot of the toolbox for prioritizing traffic calming strategies.

In this toolbox, as shown in Figure 9, two panels were designed for the purpose of comparison traffic calming strategies. In the first panel, by employing a dropdown list users are enabled to compare traffic calming strategies with respect to the criteria which have been described in Section 4.5.3. For example in Figure 9, when speed reduction is selected from the dropdown list, a diagram related to the comparison of traffic calming strategies with respect to the speed reduction criterion is displayed. According to the information provided, implementing vertical deflections is the most effective way to reduce traffic speed in residential streets. In the second panel, two radio button controls are provided. The first button is designed to display the diagram related to the normalized weights of criteria and the next is dedicated to display the diagram related to the composite priority weights of traffic calming strategies.

## 5. Evaluation of the System

Evaluation of expert systems is an important task for system developers [31, 32]. Evaluation of this expert system was conducted through the verification of the system, validation process, and comparison with similar works as follows.

### 5.1. Verification

Verification of the expert system aims to check that the expert systems works as intended without any errors. Before using expert systems, they must be verified in order to investigate whether the system is consistent and whether or not it is stable [31, 33]. Verification of the system was carried out by questioning two groups of experts. The first group consisted of three computer professionals who are highly skilled in computer science and can give recommendations or comments toward the improvement of the system. The second group consisted of three domain experts with more than 10 years of experience. For verifying the system, two different questionnaires were distributed among them. In these questionnaires, the computer experts and domain experts were asked to rank the parameters in the questionnaires according to the 5 point Likert Scale (1 representing strong disagreement to 5 representing strong agreement). The parameters and results of the verification are represented in Table 7. Results show that the average of answers is higher than 4 (agree and strongly agree) which means that the experts were satisfied.

In addition to the verification, the feedback collected from the end-users who analysed the system (such as online users) show that they are satisfied with both importance and performance of the systems, but they also made some useful suggestions for improvement. Thus, in order to increase user acceptance of the system, some modifications and adjustments were carried out in the system including: redesigning the menus and the format of web-pages, changing the size and type of fonts, increasing the use of hyperlinks (in order to access subjects faster than before), putting items in tables, and adding more images in different parts of the system that can enhance the learning process.

### 5.2. Validation

Validation of expert systems aims to confirm that the results derived from the system are compatible with the opinions of domain experts with regard to identical problems and situations. Validation of expert systems can ensure end-users of the reliability and credibility of systems during their decision making process. This process can also help developers or skilled engineers measure the accuracy of their knowledge base. In this study, the validation process was carried out for the three main modules of the expert system.

To perform the validation, three domain experts who are different from the experts who participated in the development of the expert system were asked to specify their experience and knowledge of the function (the purpose of implementing specific traffic calming measures), suitable place of implementation (local or collector streets), and the design process of the measures (in order to compare a measure designed by the system and a similar measure designed by experts). Also they were asked to prioritize traffic calming strategies according to the criteria which were used in this study. Furthermore, they were requested to express appropriate traffic calming strategies to match the safety problems which exist in the problems and solutions module. Finally, the percentages of answers which were the same as those provided by the system were calculated as shown in Table 8. According to this table, 71% of answers (average of answers) were matched with the output of the system. The result exceeds the level of performance which is expected between 50 and 60%. Therefore, it can be concluded that the system has achieved the goal for which it has been developed.

### 5.3. Comparison with Similar Works

There are various expert systems in the field of transportation engineering but specifically in the field of traffic calming, there was the lack of development. Most expert systems are related to pavement engineering, transportation management, and intelligent transportation systems (ITS). As mentioned in the introduction section, current expert systems can deal with limited aspects of traffic calming studies. For example the function of USLIMITS and E-ASSIST can be classified into both traffic safety and transportation management, but they are unable to provide detailed recommendations for implementing, designing or assessing traffic calming measures. In this regard, USLIMITS can only propose speed limit in urban streets. E-ASSISST can provide general recommendations toward managing transportation demand and improving nonmotorized transportation. Helping engineers to design traffic calming measures, recommending the suitable strategies to deal with traffic safety problems, and enabling users to compare traffic calming strategies are the benefits of CALMSYS compared to other relates systems.

## 6. Conclusion

In this study, a web-based advisory expert system for implementing traffic calming strategies in residential streets was built, verified, and validated to assist end-users including transportation engineers, safety consultants, and students. The knowledge base of this expert system includes three useful modules; each one is capable of assisting users separately according to their needs or problems they have encountered. Using VB.NET software for building the expert system has made it globally accessible. Evaluation of the system has shown that the system is reliable and functional which can encourage end-users to employ it in their traffic calming decision making processes. Feedback from reviewers includes some minor suggestions for improvement, but most of them expressed that working with the system has boosted their skills and creativity toward solving traffic safety problems. In order to satisfy users, some improvements as well as changes in style and format of the expert system web-pages were carried out. Furthermore, the following topic may be useful and will be added to the next version for improving the effectiveness and efficiency of the system.

- With regard to uncertainty and vagueness in some topics of traffic calming studies such as the process of determining speed limit, distance between traffic calming measures, and ranking traffic calming projects, using Fuzzy logic is useful and can handle these type of problems.
- In addition to the evaluation process carried out in this research, conducting usability analysis can be worthwhile. The purpose of usability analysis is to measure the efficiency (taking less time to achieve results), the ease of use, and learnability of the system (the capability of an application to enable end-users to learn how to work with the application).
- Developing traffic calming strategies to enhance the performance of the system including adding new measures to the system, modifying current measures, and updating designing process.
- Incorporating other languages such as Persian, Bahasa Melayu, and Turkish to the system to attract more users.
- Integrating the expert system with databases such as Microsoft SQL in order to store street data such as safety problems and implemented strategies.

## Figures and Tables

**Table 1:** List of major sources used in knowledge acquisition.

Number	Title	Year	Publisher
1	A Policy on Geometric Design of Highways and Streets [21]	2011	AASHTO
2	The Handbook of Road Safety Measures [22]	2009	Emerald
3	Manual on Uniform Traffic Control Devices	2003	FHWA
4	Traffic Calming: State of the Practice [4]	1999	FHEA
5	TDM encyclopedia [23]	1999	VTP
6	Alaska Traffic Calming Manual	2001	DOWL
7	Pennsylvania Traffic Calming Manual	2001	PDOT
8	Traffic Calming for Bus Routes	2005	TFL
9	International Approaches to Bicycles and Pedestrian Facility Design [24]	2006	FHWA
10	A Guide to Managing Truck Traffic on Local Streets [25]	1985	PVPC

**Table 2:** Classification of traffic calming measures.

Strategies	Measures
Vertical deflections	Speed bumps, speed humps, speed tables, speed cushion, rumble strips, and raised crosswalks/intersections
Horizontal deflections	Chicane, lateral shift, central chicane, and traffic circle
Narrowing	Choker, neck-down, road-diet, sidewalk widening, pedestrian refuge island, hatched marking, turn lane, and median
Pavement treatment	Brick paving, stone paving, and colored surface
Parking management	Parking restriction/prohibition, nonparallel parking
Volume control	Half closures, full closure, diagonal diverters, and turn prohibition
Streetscaping	Street furniture, tree planting, and gateway
Changes in speed limit	School zone, speed limit reduction, and truck speed limit
Enforcement	Police enforcement, increased punishment, and speed cameras
Special zones	Truck exclusion zone, shared space, pedestrian zone, and school zone
Traffic signs	Warning signs, regulatory signs, school signs, bicycle signs, pedestrian signs, residential signs, truck signs, special zone signs, and traffic calming signs
Improvement of street infrastructure	Crosswalk, sidewalk, bike lane, and street lighting
Network analysis	Changing street direction from one-way to two-way (or vice versa) and changing direction of a one-way street

**Table 3:** Results from the questionnaire about the importance of the parameters.

Category	Parameters	Group 1		Group 2		Group 3		*P* value
		Mean	SD	Mean	SD	Mean	SD	
Traffic parameters	Speeding in urban streets	3.80	0.45	4.60	0.55	4.40	0.55	0.074
	Through traffic	3.60	0.55	4.20	0.84	4.20	0.45	0.262
	Accident rate	4.20	0.84	3.80	0.84	4.00	0.71	0.735
	Parking occupancy	4.20	0.84	4.40	0.55	4.00	0.70	0.679
	Heavy vehicle	4.40	0.55	4.00	0.70	4.00	0.70	0.557

Geometric design parameters	Width of streets	4.00	0.70	4.40	0.55	4.40	0.55	0.503
	Length of streets	4.20	0.84	4.00	0.70	4.60	0.55	0.420
	Grade	4.20	0.45	4.20	0.84	4.20	0.84	1.000
	Curves	4.40	0.55	4.00	0.71	4.40	0.55	0.503

Infrastructure parameters	Sidewalk	3.80	0.84	4.20	0.84	4.40	0.55	0.462
	Bike lane	4.00	0.70	4.20	0.84	4.20	1.10	0.921
	Crosswalk	4.20	0.84	4.60	0.55	4.00	1.00	0.516
	Intersection	4.20	0.84	3.80	0.84	4.20	0.84	0.691
	Street lighting	4.00	0.71	4.60	0.55	4.20	0.84	0.420
	Bus stops	4.00	0.00	3.80	0.84	4.40	0.55	0.284

Land-use parameters	Density	4.20	0.84	4.40	0.55	4.60	0.55	0.641
	School	4.40	0.89	3.60	0.89	4.00	0.70	0.351
	Trip generators	4.00	0.70	3.80	0.84	4.00	1.00	0.914
	Residential complexes	4.20	0.84	4.40	0.55	4.60	0.55	0.641

**Table 4:** Conditions of traffic safety parameters.

Traffic safety parameters	Condition
Traffic speed	Speed differential more than 8 km/h
Through traffic	Thru traffic more than 50% of total traffic
Accident rate	Number of accident resulted from speeding more than three per year
Parking occupancy	Parking occupancy more than 50% in narrow street
Heavy vehicle	Heavy vehicle volume more than 25% of total traffic

Width of streets	More than one on each direction or the width of traffic lanes more than 3 meters
Length of streets	Unimpeded length more than 200 m in local streets and 300 m in collector street
Grade	Grade more than 8%
Horizontal or vertical curves	Sharp curves or Visibility problems

Sidewalk	No sidewalk or usable shoulders
Bike lane	No bike lane in street with cycle volume more than 200 per day
Crosswalk	Uncontrolled crosswalk with pedestrian volume more than 200 per day
Intersection	Uncontrolled intersections with speeding problems
Street lighting	No street lighting
Bus stops	Existence of bus stops

Density	Residential areas with more than 90 dwelling units per hectare
School	Existence of primary schools or kindergartens
Trip generators	Existence of more than three major trip generators or commercial streets in 300 m length of a street
Residential complexes	Lack of safety measures

**Table 5:** Solutions for dealing with traffic safety problems.

Problems	Solutions												
	Vert. def.	Hor. def.	Narrowing	Pavement treat.	Parking manag.	Volume control	Streetscaping	Spd. limit chang.	Enforcement	Special zones	Traffic signs	Network analysis	Infrast. develop.
Speeding	∗	∗		∗		∗	∗		∗	∗	∗	∗	
Thru Tr.	∗	∗				∗					∗	∗	
Accident	∗	∗		∗		∗	∗		∗	∗	∗		
Parking	∗		∗		∗			∗			∗		∗
Trucks								∗			∗		∗
Width			∗		∗							∗	∗
Length	∗	∗											
Grade	∗			∗				∗		∗	∗	∗	
Curves	∗				∗			∗			∗		∗
Sidewalk	∗			∗	∗			∗			∗		∗
Bike lane	∗	∗						∗			∗		∗
Crosswalk	∗		∗	∗	∗						∗		∗
Uncon. inter.	∗	∗	∗	∗	∗						∗		∗
St. lighting	∗			∗				∗			∗		∗
Bus stops	∗		∗	∗	∗						∗		∗
Density			∗	∗			∗	∗		∗	∗		∗
School	∗		∗		∗			∗	∗	∗	∗		∗
Trip gen.	∗	∗	∗	∗			∗	∗		∗	∗		∗
Res. complex	∗			∗			∗	∗			∗		

**Table 6:** A screenshot of the traffic calming strategies page.

Strategy	Measure	Description
Vertical deflection	Speed bump	Making slow points on residential complexes
	Speed hump	Making slow points on roadways
	Speed table	Making slow points on roadways
	Speed cushion	Making slow points on roadways suitable for bus routes
	Rumble strip	Alerting unaware drivers to the changes in traffic condition and environment
	Raised Crosswalk/intersection	Enforcing drivers to give way to pedestrians at crosswalks to intersections

Horizontal deflection	Chicane	Making slow points on roadways
	Lateral shift	Making slow points on roadways
	Center island Chicane	Making slow points on roadways
	Traffic circle	Making slow points at intersections

Narrowing	Choker	Reducing the width of roadways at midblock locations
	Bus bulb	Curb extension to improve the safety of passengers
	Neck-down	Reducing the width of roadways at intersections
	Road diet	Reducing the number of lanes and the effective width
	Sidewalk widening	Widening sidewalk to improve pedestrian activities
	Pedestrian refuge island	Improving the safety of pedestrian when crossing wide streets
	Hatched markings	Reducing the effective width of roadways
	Turn lane	Assigning a turn lane in the middle of roadways to reduce effective width of roadways
	Median	Constructing raised median to reduce the effective width of roadways

Pavement treatment	Pavement treatment	Using alternative materials to alert drivers to changes in traffic condition and environment

Parking management	Parking Restriction/prohibition	Restricting or prohibiting on-street parking to improve the safety of pedestrians and other vulnerable road-users
	Nonparallel parking	Different types of parking to reduce the effective width of roadways

Volume control	Full closure	Closing streets to thru traffic
	Half closure	Closing one-half of roadways
	Diagonal diverter	Managing streets to discourage thru traffic
	Turn prohibition	Closing streets at particular times to discourage thru traffic

Streetscaping	Street furniture	Enhancing the social aspect of streets
	Tree planting	Enhancing the environmental aspect of streets
	Gateway	Inform drivers about changes in traffic zone

Changes to speed limit	Speed limit reduction	Reducing current speed limit
	Heavy vehicle speed limit	Different speed limits for trucks
	School zone	Reducing speed limit at school times

Network Analysis	Converting one-way to two-way	Reducing effective width of streets and encouraging drivers to lower their speed
	Changing direction of streets	Managing streets to discourage thru traffic

**Table 7:** Experts' responses to the system verification questions.

Questions for computer professionals	Scores					
	1	2	3	4	5	Av.
(1) The user interface is user friendly				*√* *√*	*√*	4.3
(2) The system is easy to use				*√*	*√* *√*	4.67
(3) The system runs commands quickly				*√*	*√* *√*	4.67
(4) The system has no bugs				*√* *√*	*√*	4.3
(5) The system has correct codes				*√* *√* *√*		4
(6) Access to different parts of the system is easy				*√*	*√* *√*	4.67

Questions for domain experts	Scores					
	1	2	3	4	5	Av.

(1) The user interface is user friendly				*√*	*√* *√*	4.67
(2) The system is easy to use				*√* *√*	*√*	4.3
(3) The system runs commands quickly				*√* *√*	*√*	4.3
(4) The problems are well defined				*√*	*√* *√*	4.67
(5) The solutions are clear				*√* *√*	*√*	4.3
(6) Strategies are well organized				*√*	*√* *√*	4.67
(7) Measures are well described				*√*	*√* *√*	4.67
(8) The whole system is useful				*√*	*√* *√*	4.67

**Table 8:** Summary of percentage of similarity between evaluators' responses and those of the system.

Items	Similarity percentage
Function of TC measures	75%
Suitable places for TC measures	85%
Design process of TC measures	65%
Prioritization of TC strategies	60%
Solutions for safety problems	70%

Average	71%
